# Controlled‐Atmosphere Flame Fusion Single‐Crystal Growth of Non‐Noble fcc, hcp, and bcc Metals Using Copper, Cobalt, and Iron

**DOI:** 10.1002/anie.201915389

**Published:** 2020-05-08

**Authors:** Fabian M. Schuett, Derek Esau, K. Liam Varvaris, Shelly Gelman, Jonas Björk, Johanna Rosen, Gregory Jerkiewicz, Timo Jacob

**Affiliations:** ^1^ Institute of Electrochemistry Ulm University Albert-Einstein-Allee 47 89081 Ulm Germany; ^2^ Department of Chemistry Queen's University 90 Bader Lane Kingston Ontario K7L 3N6 Canada; ^3^ Department of Physics, Chemistry and Biology, IFM Linköping University 58183 Linköping Sweden; ^4^ Helmholtz-Institute-Ulm (HIU) Electrochemical Energy Storage Helmholtzstr. 11 89081 Ulm Germany; ^5^ Karlsruhe Institute of Technology (KIT) P.O. Box 3640 76021 Karlsruhe Germany

**Keywords:** computational chemistry, controlled-atmosphere flame fusion, crystal growth, single crystals, Wulff construction

## Abstract

The growth of noble‐metal single crystals via the flame fusion method was developed in the 1980s. Since then, there have been no major advancements to the technique until the recent development of the controlled‐atmosphere flame fusion (CAFF) method to grow non‐noble Ni single crystals. Herein, we demonstrate the generality of this method with the first preparation of fcc Cu as well as the first hcp and bcc single crystals of Co and Fe, respectively. The high quality of the single crystals was verified using scanning electron microscopy and Laue X‐ray backscattering. Based on Wulff constructions, the equilibrium shapes of the single‐crystal particles were studied, confirming the symmetry of the fcc, hcp, and bcc single‐crystal lattices. The low cost of the CAFF method makes all kinds of high‐quality non‐noble single crystals independent of their lattice accessible for use in electrocatalysis, electrochemistry, surface science, and materials science.

## Graphical abstract

**Grow, little bead**: Herein, we state the first growth of Cu, Co, and Fe single crystals prepared using the controlled‐atmosphere flame fusion method. The results demonstrate the transferability and potential of this method, showing that it can be used to grow single crystals of various oxygen‐sensitive metals in an effective and non‐expensive way.

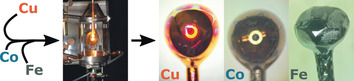

## Introduction

Non‐noble transition metals are widely used in a variety of fields, such as (electro‐)catalysis, fuel‐cell development, electrode materials for battery systems, electronic devices, and corrosion science, just to name a few.^1^, ^2^, ^3^, ^4^, ^5^, ^6^, ^7^, ^8^, ^9^ For instance, copper is known as one of the most active electrode materials in catalysis for nitrogen and carbon oxides,^1^, ^2^, ^10^, ^11^ while cobalt is mainly used in combination with its oxides for the production of new electrode and nanostructured materials in the fields of electrocatalysis, energy storage, and material science.^3^, ^4^, ^5^, ^6^, ^12^ Furthermore, research pertaining to iron and its alloys has been centrally focused around corrosion since the 1930s.^13^ In recent years, it has become an increasingly important metal for catalysis and the use in data‐storage devices.^7^, ^9^, ^14^

While there has been considerable research involving polycrystalline Cu, Co, and Fe, little research has been conducted on monocrystalline materials of these three metals. This is, in part, due to the high cost of commercially available Cu, Co, and Fe single crystals, as the methods required to grow these single crystals rely entirely on expensive ultrahigh vacuum (UHV) techniques. This is further complicated by the difficulty of working with oxygen‐sensitive metallic crystals, where undesired oxidation or corrosion can occur when they are in contact with air or electrolytes, which damages the expensive single crystals. However, the implementation of the controlled‐atmosphere flame fusion (CAFF) method (which was previously demonstrated for Ni) has made growing in‐house non‐noble‐metal single crystals less expensive and without the need for complex UHV techniques.^15^

Despite the challenges and cost, research involving single‐crystal surfaces has revealed that many reactions are strongly dependent on the specific surface arrangement of atoms. Copper, for example, catalyzes a potential‐induced interconversion between nitrate and nitrite on Cu(100), but not on Cu(111).^11^ Furthermore, a high selectivity for the formation of ethylene during the electrochemical reduction of CO_2_ was found on Cu(100), while methane was favored on Cu(111).^16^, ^17^ In the case of cobalt, the structure of individual surfaces has a significant impact on its catalytic properties; specifically for the Fischer–Tropsch reaction, Co(0001) and Co(11$\bar{2}$ 0) are efficient catalysts, while Co(10$\bar{1}$ 2) is immediately poisoned.^18^ Similarly, iron single‐crystal surfaces have different catalytic abilities, as evidenced in the synthesis of ammonia, where Fe(111) and Fe(211) are the most active catalysts while Fe(110) is nearly inactive.^7^, ^14^ It was also found that different surfaces have different reaction rates while undergoing corrosion and passivation under varying conditions. However, there is still an ongoing discussion regarding the mechanism for the formation of monoatomic and three‐dimensional oxide films.^8^, ^13^, ^19^ Findings such as these highlight the importance of fundamental research with single‐crystal surfaces of noble‐ and non‐noble‐metal systems for heterogeneous (electro‐)catalysis. A current example for this is the recently published work of J.‐J. Shyue et al. about the flame fusion growth of copper and copper/nickel‐alloy single crystals, which is based on the results presented herein.^20^

In this work, we show the first growth and analysis of Cu, Co, and Fe single crystals prepared using the CAFF method. The high quality of the prepared single crystals was verified using scanning electron microscopy (SEM) combined with energy‐dispersive spectroscopy (EDS) and Laue X‐ray back‐scattering. Furthermore, Wulff constructions (using DFT‐calculated surface energies) were performed to evaluate the observed structures of the grown systems. These results show the generality of the method, demonstrating that it can be used to grow crystals of other oxygen‐sensitive metals in addition to the previously described growth of Ni.^15^ Hence, the CAFF method can be stated as a fast, effective, and cheap way for the in‐house growth of noble‐ and non‐noble‐metal single crystals, which further extends the scope of all single‐crystal‐preparation techniques without challenging any other technique. The discussion of advantages, disadvantages, as well as differences of the various existing single‐crystal‐preparation methods is certainly beyond the scope of the present work but could be the topic of a future extended review.

## Results and Discussion

Preparation of the three (Cu, Co, and Fe) single crystals was made possible with the CAFF method. This method is an improvement of the flame fusion method originally developed by Clavilier in the 1980s,^21^ where a metal wire was melted in a hydrogen‐oxygen flame. The resulting liquid metal bead is then slowly cooled by lowering the flame, resulting in a bead‐shaped single crystal, where various facets of low and high Miller‐index surfaces are observed on the surface of the crystal.^21^ With the subsequent orientation and polishing of those poly‐oriented spherical single crystals (POSSCs), it is a simple and effective method to prepare in‐house monocrystalline surfaces, for example, as electrodes for electrochemical measurements.^21^ Before the recent development of the CAFF method, the growth of such POSSCs was limited to noble metals that are stable to thermal oxidation, which can occur when heating a metal, such as Pt and Au, in air.^15^ In contrast, the CAFF method now incorporates a specifically designed semi‐sealed atmosphere‐control chamber into the setup of Clavilier. Using this chamber, a slightly reducing environment is created by the implementation of a continuous argon stream while employing a hydrogen‐rich flame. Further description of this method can be found in ref. 15 as well as in the Supporting Information.

Using a force balance between the surface tension of the molten metal bead and gravity pulling the growing bead down [Eq. (1)], the maximum possible radius (*R*_max_) of the beads can be calculated by^22^, ^23^

(1) $$\frac{4}{3}\pi R_{max}^{3}\rho g=2\pi r\sigma ,$$

where *σ* is the surface tension of the molten metal (in kg s^−2^), *r* is the diameter of the starting wire, *g* is the gravitational acceleration (in m s^−2^), and *ρ* is the density of the molten metal (in kg m^−3^). Using a starting wire with a diameter of 1 mm and applying literature values to Eq. (1) for the properties of all three metals results in the maximum possible diameter (2*R*_max_) listed in Table 1.

**Table 1:** Theoretical maximum crystal diameter according to Eq. (1).

metal	lattice	*ρ* [kg m^−3^]	*σ* [kg s^−2^]	2*R*_max_ [mm]
Cu	fcc	7992^24^	1.27^24^	4.58
Co	hcp	7827^25^	1.99^26^	5.38
Fe	bcc	7035^27^	1.98^26^	5.56

### Cu: Face‐Centered Cubic

**Figure 1:**
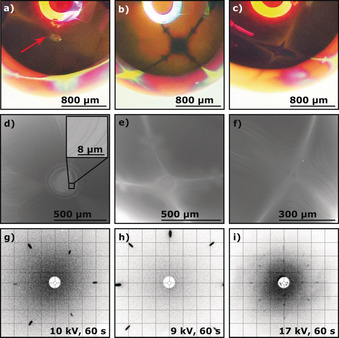
Identification and characterization of the fcc copper low‐Miller‐index surfaces Cu(111), Cu(100), and Cu(110) after slight oxidation of the bead in air. a)–c) Optical‐microscopy images and d)–f) SEM images of each surface. The red arrow in (a) indicates the Cu(111) facet and the inset in (d) shows the “wedding‐cake” surface layering of the same facet. g)–i) Corresponding Laue X‐ray back‐scattering patterns.

Preparation of copper single crystals with the flame‐fusion method leads to Cu beads that crystallize in the face‐centered cubic (fcc) lattice like platinum or nickel for which similar growth behaviors were observed. The facets on Cu are barely visible to the naked eye due to their small size and the high reflectivity of the surface. Therefore, thermal oxidation of the Cu surface in air, which was previously established for nickel POSSCs, was applied.^15^ Since oxidation rates of various surface orientations differ from one another, this approach revealed the various facets of the crystal.^11^, ^28^ Similar to nickel POSSCs, after thermal oxidation, a pure and defect‐free Cu surface could be regained through inductive annealing in a mildly reducing atmosphere.^15^ Figure 1 a–c shows optical‐microscopy images of a Cu(111), Cu(100), and Cu(110) surface, respectively, after slight oxidation in air. The low Miller‐index surfaces (basal planes) as well as the stepped surfaces appear reflective and metallic, while the rest of the crystal (kinked surfaces) formed a matte pink‐orange layer. Figure 1 d–f shows SEM images of the same facets for comparison. The SEM imaging also indicates that Cu(111) adopts a layered structure as it crystallizes (see inset of Figure 1 d). This “wedding‐cake” crystallization mechanism is also observed for other fcc POSSCs such as platinum and gold.^22^, ^29^, ^30^ Finally, Laue X‐ray back‐scattering patterns of the basal facets confirm the high quality of the grown Cu POSSCs (Figure 1 g–i). The slightly elongated spots closer to the edges of the diffraction patterns are an artifact of the used Laue detection system that arises for higher diffraction angles and not a sign for poor crystallinity. A more precise description about this can be found in the Experimental Section in the Supporting Information.

**Figure 2:**
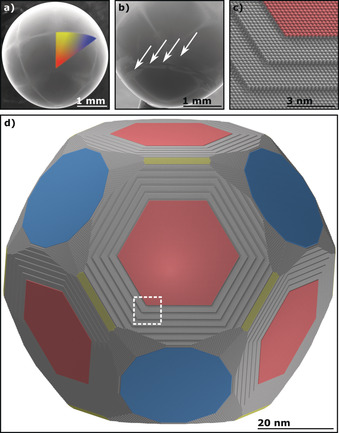
Experimentally obtained and theoretically simulated representations of the stereographic projection of a fcc copper POSSC. The colored planes indicate the low‐Miller‐index surfaces Cu(111) (red), Cu(100) (blue), and Cu(110) (yellow). a) SEM image of a slightly oxidized Cu POSSC with a color‐coordinated stereographic triangle. b) SEM image of the grain boundaries of a multi‐crystalline copper bead. The white arrows highlight each grain boundary. c) Magnification of the white rectangle in (d) that shows the surface layering of the Cu(111) facet. d) Wulff construction of a Cu single crystal particle of approximately 86.5 nm diameter.

The quality of the crystals can also be assessed through the aforementioned thermal oxidation of the Cu single‐crystal surface. The stepped and basal surfaces are connected by oxidation lines, which form the pattern of multiple fcc stereographic triangles that encapsulate the whole bead.^22^, ^28^, ^31^, ^32^, ^33^ The symmetry of these stereographic triangles around the bead is indicative of the monocrystallinity of the crystal (see Figure 2 a). In this Figure, as well as throughout the rest of this Research Article, the (111) surface is always depicted in red, while the (100) and (110) orientations are shown in blue and yellow, respectively. Any discontinuities in the stereographic triangles are evidence of multi‐crystallinity, which is demonstrated in Figure 2 b), where a multi‐crystalline bead was grown (here, the white arrows indicate different grain boundaries).

The shape of the copper beads is additionally corroborated by a Wulff construction (see Figure 2 d), which shows a similar size distribution and the same orientation of the facets as a grown single crystal. Additionally, the layered structure observed on the Cu(111) facets (see Figure 1 d and inset) is consistent with the layering of the (111) facets by the Wulff construction, as can be seen in Figure 2 c, which is a magnification of the white‐marked area in Figure 2 d. The main difference between theory and experiment is that the experimentally grown bead adopted a more spherical shape. This discrepancy could be due to the fact that all calculations were done at 0 K without taking any entropic effects into consideration.

### Co: Hexagonal Close‐Packing

**Figure 3:**
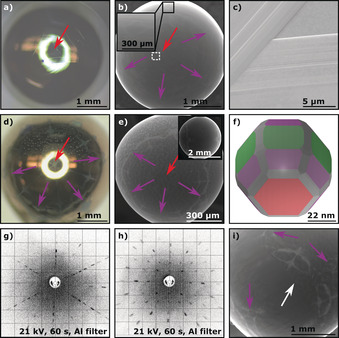
Comparison of non‐oxidized vs. oxidized hcp cobalt POSSCs as well as their characterization and theoretical representation using Laue X‐ray back‐scattering and Wulff construction. The colors indicate the basal Co(0001) (red), Co(1$\bar{1}$ 01) (purple), and prismatic Co(1$\bar{1}$ 00) (green) planes. a) Optical‐microscopy image of a Co POSSC showing the Co(0001) facet. b) SEM image of a Co POSSC showing the Co(0001) and Co(1$\bar{1}$ 01) facets, with an inset showing a magnified Co(1$\bar{1}$ 01) facet. c) Magnification of the white rectangle in (b) showing the layered growth structure of the Co(0001) facet. d) Optical‐microscopy and e) SEM image of a thermally oxidized Co POSSC showing the Co(0001) and Co(1$\bar{1}$ 01) facets. The inset in (e) shows a different perspective to indicate the hcp symmetry. f) Wulff construction of a Co single‐crystal particle of approximately 80.1 nm diameter. g), h) Laue X‐ray back‐scattering patterns of the basal Co(0001) and prismatic Co(1$\bar{1}$ 00) planes, respectively. i) SEM image of a bi‐crystalline cobalt bead (the white arrow indicates the grain boundary).

Similar to Cu, the facets present on Co single‐crystal surfaces are difficult to discern with an optical microscope, except for the Co(0001) facet that is faintly observable as a small triangle (see red arrow in Figure 3 a). SEM analysis reveals the prismatic Co(1$\bar{1}$ 01) planes being visible as six small, diamond‐shaped facets evenly distributed around the central Co(0001) facet (see purple arrows and inset in Figure 3 b). Magnification of the Co(0001) facet (white rectangle in Figure 3 b) reveals that a layered growth mechanism takes place during the crystallization of the Co(0001) facet, shown in Figure 3 c), which is similar to what is observed for the Cu(111) facet.

Thermal oxidation of the surface of the Co POSSC reveals the stereographic hcp projection. Figure 3 d shows an optical image of a thermally oxidized Co POSSC, clearly showing the central Co(0001) facet (red arrow) surrounded by six Co(1$\bar{1}$ 01) facets (purple arrows). SEM imaging of the thermally oxidized Co POSSC shows the high symmetry of the facets, which is also represented on the opposite side of the crystal, demonstrating the high quality of the crystals (see Figure 3 e and top view of the Co POSSC in the inset). Analysis of the thermally oxidized Co POSSCs shows that surface pitting, shown in Figure 3 e as small spots, can occur during the oxidation process. These rectangular pits are unique to Co and could not be observed for Cu or Ni.^15^ The shape and orientation of the hcp POSSCs were again verified by a Wulff construction utilizing DFT‐obtained surface energies, as spherical hcp single crystals of this size have never been prepared before. Figure 3 f shows the Wulff construction of a Co single‐crystal particle of approximately 80.1 nm diameter, the structure of which agrees with the newly grown Co POSSCs. In the image the Co(0001) facet is depicted in red, as it has the same surface arrangement of atoms as fcc(111) surfaces (see Figure 2 c), while the Co(1$\bar{1}$ 00) and Co(1$\bar{1}$ 01) facets are shown in green and purple, respectively. To confirm that the cobalt beads are monocrystalline, Laue X‐ray back‐scattering analysis was performed. Figure 3 g,h shows the diffraction patterns of the hexagonal Co(0001) basal plane and the prismatic Co(1$\bar{1}$ 00) plane, respectively. Similar to Cu, multi‐crystalline Co beads are easily detected by discontinuities of the stereographic projection, as shown on a bi‐crystalline bead in Figure 3 i. Here, the white arrow indicates the grain boundary and the purple arrows highlight Co(1$\bar{1}$ 01) facets.

Unlike the other metals that have been grown using traditional flame fusion or the CAFF method, cobalt exhibits more than one allotropic phase. Below 422 °C, Co adopts the hexagonal closed‐packing (hcp) ϵ‐phase, while above 422 °C, the fcc α‐phase is favored up to the melting point at 1495 °C.^34^ For Co, crystallization occurs in the fcc α‐phase temperature range, which would lead to the possibility that cobalt might crystallize and adopt the fcc lattice structure. However, as shown in the previous section, hcp Co POSSCs are always grown using standard growing conditions. This might be due to the inherent properties of the starting wire that is used for the crystal growth, since cold‐worked Co wire only contains hcp crystal grains.^35^ It might also be possible that the crystal undergoes a phase transition from fcc to hcp during cooling.

**Figure 4:**
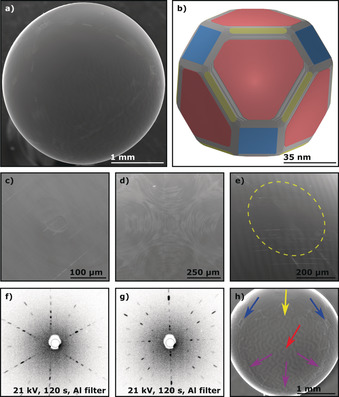
Implications of the phase transition from hcp to fcc cobalt and its effect on the surface and bulk structure of the POSSCs. a) SEM image of a cobalt single crystal with fcc oxidation patterns, grown from a thermally pre‐treated wire. b) Wulff construction of a fcc Co single‐crystal particle of approximately 100.2 nm diameter, the colored planes indicate the Co(111) (red), Co(100) (blue), and Co(110) (yellow) surfaces. c)–e) SEM images of the three low‐Miller‐index planes Co(111), Co(100), and Co(110). f), g) Laue X‐ray back‐scattering patterns of a Co crystal with fcc oxidation patterns indicating the basal Co(0001) and prismatic Co(1$\bar{1}$ 00) planes. h) Surface‐phase transitions between fcc and hcp oxidation patterns indicating growth and deformation faults, where the colored arrows indicate the Co(100) (blue), Co(110) (yellow), Co(111)/Co(0001) (red), and Co(1$\bar{1}$ 01) (purple) facets.

Repeated annealing of Co in the fcc α‐phase temperature range (above 422 °C) increases the amount of fcc grains in the material, however, because cobalt is influenced by its thermomechanical history.^35^, ^36^ The amount of fcc grains in cobalt is also dependent on the annealing temperature but not on time.^36^, ^37^ This concept was explored by annealing a starting wire repetitively just below the melting point, which gave rise to cobalt single crystals in which fcc stereographic triangles are readily identifiable after slight oxidation (see Figure 4 a). The Wulff construction of a fcc cobalt particle of approximately 100.2 nm diameter is consistent with the oxidation patterns on the surface of the crystals (see Figure 4 b). Figure 4 c–e shows magnified SEM images of the Co(111), Co(100), and Co(110) facets, respectively. However, Laue X‐ray back‐scattering revealed that even though the surface shows fcc oxidation patterns, the bulk remains hcp (shown in Figure 4 f,g for the Co(0001) and Co(1$\bar{1}$ 00) facets, respectively). As no twinning in the Laue X‐ray back‐scattering patterns can be observed, it is assumed that there may be a growth or deformation fault causing a phase shift in the near‐surface region.^36^ This assumption is supported by seamless surface‐phase transitions that were observed in a few of those crystals. This can be seen for one example in Figure 4 h, which shows a cobalt single crystal with the fcc pattern merging into the hcp pattern. The fcc and hcp patterns around the phase transition are indicated by color‐coded arrows.

### Fe: Body‐Centered Cubic

Similar to Co, Fe exhibits more than one allotropic phase between room temperature and the melting point: below 910 °C, the stable phase is the body‐centered cubic (bcc) α‐phase. Between 910 °C and 1390 °C, the lattice transforms into the fcc γ‐phase and from 1390 °C to the melting point at 1534 °C the bcc δ‐phase is most stable.^38^, ^39^, ^40^ Nevertheless, for iron, it was not possible for us to observe any fcc modification; neither before, nor after the crystal growth, nor through thermal treatment of the bcc starting wire.

**Figure 5:**
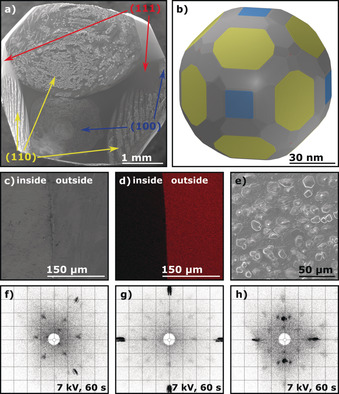
Structural characterization of bcc iron POSCs using SEM, EDS, and Laue X‐ray back‐scattering as well as a theoretical model of a particle using Wulff construction. The colored planes and arrows indicate the low‐Miller‐index surfaces Fe(111) (red), Fe(100) (blue), and Fe(110) (yellow). a) SEM image of a Fe single crystal as grown. b) Wulff construction of a Fe single crystal of approximately 89.5 nm diameter. c) Polished cross‐section of a grown Fe bead and d) the corresponding EDS oxygen mapping (red). e) SEM images of a Fe(110) plane from the top. f)–h) Laue back‐scattering patterns of the Fe(111), Fe(100), and Fe(110) planes, respectively.

Contrary to other metals grown via the traditional flame fusion or CAFF method, iron does not grow spherically.^15^, ^22^ This can be seen in Figure 5 a, which shows a SEM image of an iron single‐crystal bead. Hence, the term POSSC does not seem to uniformly fit all single crystals made with a flame fusion technique and it might be better to use the more general term poly‐oriented single crystal (POSC). Due to its initial shape, no surface treatment needs to be conducted, as the facets are readily identifiable. Figure 5 b shows the corresponding Wulff construction of an iron particle of approximately 89.5 nm diameter. Unlike the fcc crystal structure, the (111) surface is the least favored of the three basal facets in the bcc lattice, which results in a very low representation on the surface (see Figure 5 a,b). In the case of copper and cobalt as well as our previous results for nickel, the Wulff constructions overestimated the size of the facets compared to the grown POSSCs.^15^ In the case of iron, there is a closer resemblance between theory and experiment, with the Wulff construction even slightly underestimating the relative size of the low Miller‐index facets compared to the experiments. The calculations corroborate the experiments in that the Fe(110) facet is dominating, while Fe(111) is hardly visible at all.

The SEM images show that the surface of the iron POSC is not smooth or completely metallic. Even in the mildly reducing atmosphere created through the implementation of the CAFF method, magnetite forms as a product of the iron‐catalyzed water‐splitting reaction taking place at temperatures above 350 °C. In this reaction, the water produced by the flame dissociates into hydrogen and oxygen that directly forms ferrous oxide and is then further oxidized to magnetite.^41^ When the cooling of the bead starts, the magnetite separates from the iron phase and crystallizes on the outside (mainly on the top near the wire) of the Fe crystal. At this point, one can already observe the shape which the crystal is adopting. To verify that Fe_3_O_4_ only forms a thin layer on the surface of the iron single crystals, the beads were cut to produce cross‐sections that were subsequently analyzed using SEM and EDS. A SEM image and corresponding elemental EDS mapping in which the red color indicates oxygen are shown in Figure 5 c,d. As there is not enough magnetite to completely coat the top of the bead, elemental iron is slightly exposed at the surface of the bead, which results in a more roughened surface morphology (see Figure 5 e). Furthermore, Laue X‐ray back‐scattering measurements were conducted to verify the monocrystallinity of the Fe POSCs below the magnetite layer. The patterns for the three low Miller‐index surfaces Fe(111), Fe(100), and Fe(110) are shown in Figure 5 f–h. Agreement between the Laue X‐ray back‐scattering patterns with previously recorded low‐energy electron diffraction (LEED) patterns of clean iron surfaces confirm that the Fe beads adopt the bcc lattice structure.^42^ The Laue X‐ray back‐scattering patterns also reveal a double feature at the most intense reflections. These reflections were found to correspond to magnetite and indicate that the magnetite layer around the iron beads is also monocrystalline.

**Figure 6:**
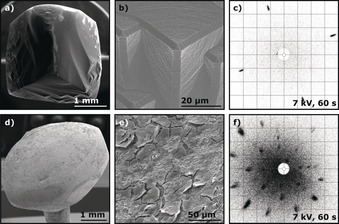
SEM images and Laue back‐scattering patterns comparing a magnetite and an etched iron single crystal. a) SEM image of a magnetite single crystal. b) Magnification of the triangles at the side. c) Laue back‐scattering pattern of the (100) plane of the magnetite single crystal. d) SEM image of an etched iron single crystal. e) Magnification of the acid‐roughened surface and f) Laue back‐scattering pattern of the Fe(100) plane.

Applying the CAFF method, it is not possible to grow an iron single crystal without a magnetite surface layer due to the unavoidable presence of water vapor created by the hydrogen–oxygen flame. However, the oxygen content in the hydrogen–oxygen flame has a significant impact on the thickness of the magnetite around the bead. A larger ratio of oxygen in the flame increases the amount of magnetite (that is, the thickness of the shell) and may eventually result in a complete magnetite single crystal (see Figure 6 a–c). However, a lower proportion of oxygen makes it challenging to melt the wire evenly, as the flame is colder and results in an unstable melt that causes improper crystallization. In any case, one can directly observe the change of the shape of the “bead” with different amounts of oxygen in the flame and thus different quantities of magnetite in the iron melt.

The growth of the iron single crystals also reveals the limits of the CAFF method; metals that react with water vapor at high temperatures will always form an oxide during the crystallization process. The method works for Fe, as the melting points of iron (1538 °C^27^) and magnetite (1597 °C^43^, ^44^) are quite similar. If the melting points of the pure metal and the corresponding oxide(s) differ significantly, or if the starting metal is even more reactive with water vapor than iron, it is expected that the CAFF method will no longer produce metallic single crystals.

For many applications, cut and polished single crystals are required; therefore, a magnetite surface layer is usually inconsequential. However, for certain measurements, it may be desirable to remove the magnetite coating and expose metallic iron. This can be achieved by cooling the iron single crystals in a hydrogen stream, thereby reducing the magnetite layer to some mixed iron oxide. This oxide can then be etched away using concentrated HCl or diluted H_2_SO_4_, resulting in a metallic but roughened iron surface (see Figure 6 d–f).

## Conclusion

In this work, we have shown the first successful application of the controlled‐atmosphere flame fusion (CAFF) methodology to grow fcc Cu, hcp Co, and bcc Fe single crystals. Their quality was verified using scanning electron microscopy and Laue X‐ray back‐scattering. To gain further insights into the shape and surface distribution of the different exposed facets, Wulff constructions were performed and compared with the newly prepared crystals. In the case of Co and Fe, their allotropy and the associated implications on growth and annealing were discussed as well. As outlook and improvement, one could track the cooling curves with thermal cameras to gain more quantitative information about the cooling and solidification rates during the crystal‐growth process.

These results provide the first poly‐oriented single crystals of hexagonally close‐packed and body‐centered cubic crystal structures. This allows for a simple production and study of monocrystalline surfaces for both low and high Miller‐index surfaces. Ready identification of grain boundaries and multi‐crystallinity allows for studies in interfacial crystallography on easily defined crystal boundaries. We believe this development will allow an expansion of research not only in materials science but additionally in the areas of heterogeneous catalysis, electrocatalysis, as well as interfacial electrochemistry and surface science.

## Conflict of interest

The authors declare no conflict of interest.

## Supporting information

As a service to our authors and readers, this journal provides supporting information supplied by the authors. Such materials are peer reviewed and may be re‐organized for online delivery, but are not copy‐edited or typeset. Technical support issues arising from supporting information (other than missing files) should be addressed to the authors.

Supplementary
