# Supplementary material for: Controlled‐Atmosphere Flame Fusion Single‐Crystal Growth of Non‐Noble fcc, hcp, and bcc Metals Using Copper, Cobalt, and Iron
Source: Angew Chem Int Ed Engl. 2020 May 8;59(32):13246–52. doi: 10.1002/anie.201915389 (PMC7496678; doi:10.1002/anie.201915389)
Supplement: Supplementary file 1 — Supplementary [file ANIE-59-13246-s001.pdf]

Angewandte Chemie
Eine Zeitschrift der Gesellschaft Deutscher Chemiker

Supporting Information

Controlled-Atmosphere Flame Fusion Single-Crystal Growth of Non-Noble fcc, hcp, and bcc Metals Using Copper, Cobalt, and Iron

Fabian M. Schuett[+], Derek Esau[+], K. Liam Varvaris, Shelly Gelman, Jonas Björk, Johanna Rosen, Gregory Jerkiewicz, and Timo Jacob**

anie_201915389_sm_miscellaneous_information.pdf

Supporting Information

Table of Contents

Experimental Procedures...2

Crystal Preparation...2

Thermal Oxidation...3

Imaging...3

Laue X-ray back-scattering...3

Computational Details...4

Results and Discussion...5

Table S1...5

Table S2...6

Table S3...7

Table S4...8

References...9

Author Contributions...9

Experimental Procedures

Crystal Preparation

The bead-shaped, poly-oriented spherical single crystals (POSSCs) of Cu (Alfa Aesar, Puratonic grade 99.9999% in purity) and Co (Alfa Aesar, Puratonic grade 99.995% in purity) and, single crystals of Fe (Alfa Aesar, Puratonic grade 99.995% in purity) were grown from a starting wire being 1.0 mm in diameter. All wires were degreased in hot acetone (Fisher Scientific, ACS Reagent) under reflux for 4 h using a Soxhlet extractor prior to crystal growth. The gases used to generate the hydrogen-oxygen flame with a micro-torch (The Little Torch, Smith Equipment) were of ultra-high purity (UHP) H_2(g) and O_2(g) (Praxair, 5.0 and 4.3 in purity, respectively). All gas flow rates were controlled with gas flow meters (Omega and Swagelok). The linear actuator (Zaber Technologies) used to move the torch, thereby to control the cooling rate of the crystal, was placed on a stainless-steel assembly connected to linear tracks (Optikon Corporation) to allow for *x* and *y* movements for optimal alignment of the flame. The wire was held in place by a ceramic tube (yttria-stabilized zirconia, OMEGATITE 450, Omega) and projected onto a white screen with optical lenses (Ektanar, Kodak) for the melting of Co and Fe.[1] Due to the low melting point of Cu and the resulting low intensity light that is emitted during melting, the crystal growth was monitored directly with a large, magnifying lens.

The principles of the flame fusion methodology used to grow noble metal single crystals and the recent development of the controlled-atmosphere flame fusion (CAFF) technique to grow Ni POSSCs are described in detail elsewhere.[1,2] However, a summarized description of the CAFF method is described in the following section:

The main component of the CAFF set-up is a custom-made semi-sealed chamber consisting of a stainless-steel bottom plate with gas inlets and outlets and a quartz glass cone (see Figure S1). The gas inlets allow for a gentle, yet uniform stream of UHP Ar(g) (Praxair, 5.0 in purity) to fill the chamber without disturbing the flame. The stream of Ar(g) removes ambient oxygen from the chamber, allowing the oxygen-sensitive metals to crystallize in a slightly reducing atmosphere. The open chamber top allows for the hot gases and water vapour to be expelled from the chamber while allowing for the ceramic holder to be inserted into the chamber. The transparency of the quartz allows for the solid/melt interface to be monitored while maintaining the slightly, reducing atmosphere.

The relevant modifications for the growth of Cu, Co and Fe single crystal is described below:

Due to the variation in melting points, varying flow rates for the hydrogen-oxygen flame were used for each metal. The following flow rates were determined using many trials and gave the best results for our system. It is important to mention that the optimal flow rates will vary, depending on the size of the chamber, the diameter of the torch-tip and the diameter of the starting wire. For Cu, the flow rates of H_2(g) and O_2(g) were 1.0 L min^{-1} and 0.15 L min^{-1}, respectively. The flow rates used for Co of H_2(g) and O_2(g) were 1.0 L min^{-1} and 0.20 L min^{-1}, respectively. Lastly, the flow rates used for Fe were 1.0 L min^{-1} and 0.22 L min^{-1} for H_2(g) and O_2(g), respectively. The flow rate of Ar(g) being introduced into the chamber was 5.0 L min^{-1} and the diameter of the torch-tip was 2 mm for all three metals.

During flame fusion single crystal growth, the atoms must be given enough time to arrange themselves into a monocrystalline lattice, which depends on distance the torch needs to move and the rate at which it is moved. Therefore, the two main growth parameters, that need to be considered, are the rate at which the torch is lowered in µm s^{-1} and the total distance the torch is moved with the linear actuator in mm during the crystal growth. The time required appears to be unique to each metal, which is why these two metrics are important. Copper crystallizes very readily; from start of the crystallisation process to the end, the torch is only moved between ca. 1.5 - 2.5 mm and as a result, we chose a rather slow cooling rate of 1 µm s^{-1}. Cobalt was found to behave similarly to Copper, with a torch movement of 2.5 – 3.0 mm. Therefore, we also chose a cooling rate of 1 µm s^{-1}. Due to the iron-catalysed water splitting reaction, which produces more magnetite the longer the Fe is exposed to the flame, it was crucial to conduct the crystallisation much quicker. Hence, iron is a bit of an exception and the cooling rate parameter is of much higher importance than the total distance. The effect of the magnetite formation can directly be observed with different cooling rates: with a rate of 1 µm s^{-1} the growth takes longer, so the result is a crystal with a complete magnetite coating. The paper presented iron beads with a thin and incomplete magnetite coating, and metallic iron appearing on the surface (Figure 5), which were achieved with a cooling rate of 5 µm s^{-1}.

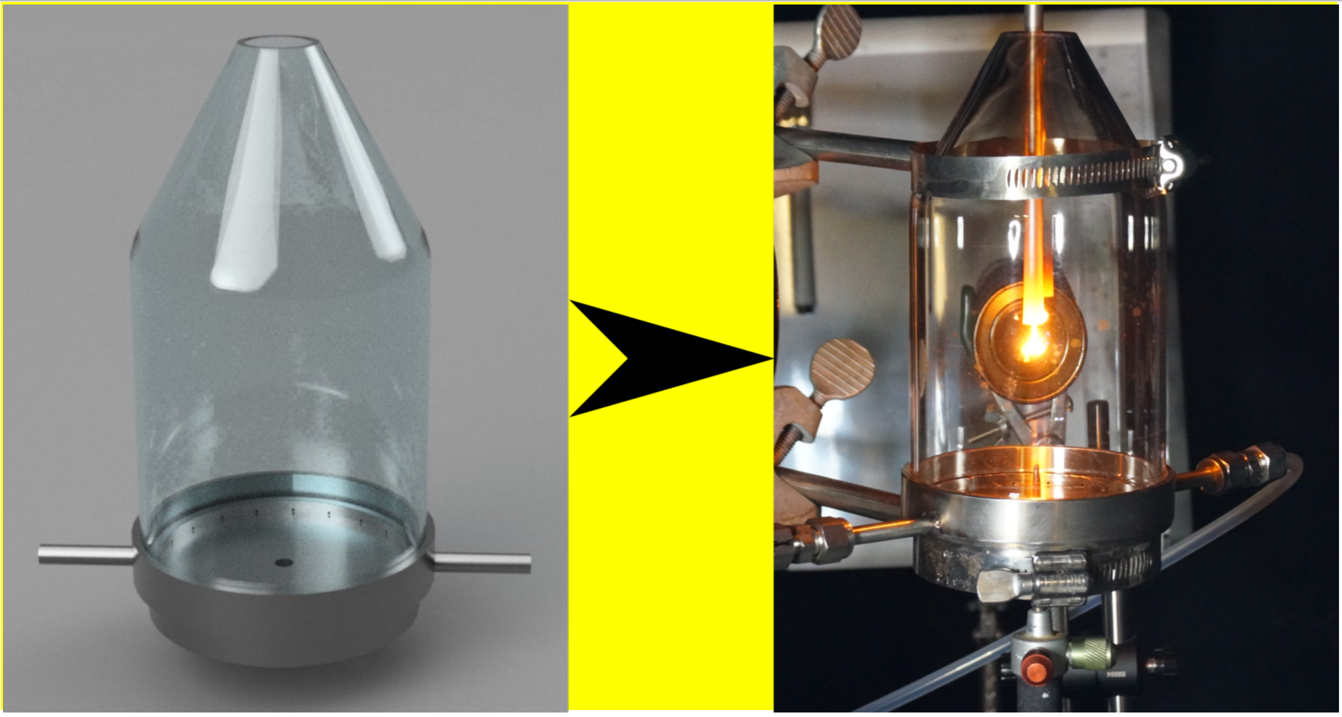

Figure S1. Left: computer-generated technical drawing of the chamber parts; the quartz glass cone and the stainless steel bottom plate with the two gas inlets on the outside and the circular arranged gas outlets on the inside. The bottom hole is used as inlet for the torch, while to top opening is the inlet for the metal wire mounted in a ceramic holder. Right: Optical image of the CAFF set-up growing a Co single crystal.

Thermal Oxidation

In order to ease the identification of facets on the Cu and Co POSSCs, slight thermal oxidation in air is a simple and effective method to achieve this. The single crystal should be positioned quite far away from the micro-torch (the distance depends on the torch size and the flow rates of the gases) such that it does not heat up too fast when the flame is ignited. With our design, we could achieve the best results by placing the beads approximately 1 cm above the chamber exposed to air. The gas flow rates of the hydrogen-oxygen flame were 0.9 L min^{-1} and 0.1 L min^{-1} for hydrogen and oxygen, respectively. The time exposed to the top of the chamber depends on the desired level of thermal oxidation.

Imaging and Graphic Design

Scanning electron microscopy (SEM) measurements were conducted using a Quanta 250 FEG instrument, FEI, equipped with a tungsten filament emission source. The single crystals were placed in a SEM holder (Ted Pella) for imaging. Energy Dispersive Spectroscopy measurements were taken with an EDAX Element EDS system. The electron beam was operated at 20 kV. Optical microscopy images were acquired using a LEICA S4E light microscope equipped with a digital camera (Sony α58) for image acquisition. Technical drawings and three-dimensional graphics presented in this work were rendered using Autodesk® Fusion 360™, version v.2.0.5688 from April 23, 2019.

Laue X-Ray Back-scattering

The Laue X-ray back-scattering measurements were conducted with 25 kV and 20 mA employing an X-ray generator (Seifert Iso-Debyeflex (ID) 3003 with a tungsten anode) connected to a Multiwire Real-Time Back-Reflection Laue Camera System (model MWL120). The detector uses Ar ions that have become ionized by the back-scattered X-rays to create a pattern based on the charge

measured by ions as they impact the proprietary electronics at the back of the detector. The back-scattering pattern is then generated using proprietary software (Northstar 7). For samples that fluoresce, a 0.5 mm aluminum filter was used. The settings used to generate each Laue back-scattering pattern is labeled on the images in the main text, as well as if the Al filter was used.

The Multiwire detector measures backscattered X-rays in real-time. Therefore, it uses an array of horizontal and vertical wires to measure incoming argon ions that have been ionized by the backscattered X-rays coming from the crystal. Due to the internal geometry of the detection wires, the spots at higher diffraction angles get slightly elongated as there is a slight time delay when they are measured, compared to those closer to the center. Therefore, the spots in the middle (lower diffraction angles) are more round then the ones closer to the edges.

Computational Details

Surface energies entering the Wulff construction were calculated within the framework of density functional theory (DFT) using the VASP code[3] by employing a planewave basis set expanded to a kinetic energy cut-off of 350 eV and projector-augmented wave method to describe ion-core interactions.[4] Exchange-correlation effects were described by the Perdew–Burke–Ernzerhof (PBE) functional[5] and in all calculations spin-polarization was included. Different number of surface layers were used for the calculations of the various surface facets and was tested for convergence, and as a role of thumb the slab thickness was more than 20 Å. Surface energies are numerically converged within 0.01 J/m^2. Top and bottom of slabs of periodic images were separated by more than 14 Å of vacuum region. The convergence with respect to k-point sampling was tested for the (111)-surface and was adjusted depending on the surface unit cell for each surface to ensure equal density of ***k***-points as for the (111)-facet. All structures were geometrically optimized until the forces on all atoms were smaller than 0.01 eV/Å. The specific computational settings for all surfaces are given in Tables S1-S4. Wulff constructions were created using the implementation in the Atomic Simulations Environment.[6]

Results and Discussion

Table S1. Information about the calculations of different *fcc* Cu surfaces, including the number of layers for respective surface, as well as the size of each surface in terms of vertical distance between top and bottom atom of the slab (z_{max} - z_{min}), number of ***k***-points, and surface energy. For the Wulff construction we used the surface energies obtained for relaxed systems.

	# of layers	z_{max} - z_{min} (Å)	*k*-point sampling	Surface energy (J/m²)
**Cu(111)**	11	20.99	20x20	1.317
**Cu(665)**	114	20.85	2x20	1.355
**Cu(554)**	94	20.81	2x20	1.359
**Cu(443)**	76	21.29	3x20	1.382
**Cu(332)**	56	21.31	3x20	1.416
**Cu(221)**	36	21.20	6x14	1.460
**Cu(331)**	26	20.85	10x10	1.520
**Cu(551)**	42	20.87	6x12	1.528
**Cu(110)**	24	29.56	20x14	1.535
**Cu(320)**	42	20.16	4x14	1.611
**Cu(210)**	28	21.95	6x20	1.592
**Cu(310)**	38	21.27	9x14	1.583
**Cu(410)**	48	20.72	4x14	1.570
**Cu(510)**	60	21.03	6x14	1.549
**Cu(610)**	72	21.21	2x14	1.532
**Cu(100)**	14	21.81	20x20	1.440
**Cu(511)**	31	20.99	6x20	1.527
**Cu(311)**	20	20.82	9x20	1.531
**Cu(211)**	30	20.78	6x20	1.493
**Cu(322)**	50	21.16	4x20	1.427
**Cu(433)**	70	21.51	3x20	1.399
**Cu(544)**	88	20.94	2x20	1.359
**Cu(321)**	44	20.89	9x20	1.556
**Cu(431)**	60	21.03	4x9	1.563
**Cu(432)**	64	21.26	3x12	1.528
**Cu(531)**	35	20.89	6x9	1.613
**Cu(654)**	102	20.92	2x12	1.429
**Cu(731)**	45	20.82	4x9	1.527
**Cu(951)**	60	20.73	3x6	1.580

Table S2. Information about the calculations of different *hcp* Co surfaces, including the number of layers for respective surface, as well as the size of each surface in terms of vertical distance between top and bottom atom of the slab (z_{max} - z_{min}), number of ***k***-points, and surface energy. For the Wulff construction we used the surface energies obtained for relaxed systems.

	# of layers	z_{max} - z_{min} (Å)	*k*-point sampling	Surface energy (J/m²)
Co(0001)	11	22.12	20x20	2.119
Co($1\bar{1}08$)	84	20.24	2x20	2.388
Co($1\bar{1}06$)	64	20.06	3x20	2.443
Co($1\bar{1}04$)	46	20.35	5x20	2.507
Co($1\bar{1}03$)	36	20.68	6x20	2.514
Co($1\bar{1}02$)	30	21.08	8x20	2.545
Co($2\bar{2}03$)	50	20.30	5x20	2.530
Co($3\bar{3}04$)	36	20.46	3x20	2.509
Co($1\bar{1}01$)	24	21.22	11x20	2.429
Co($2\bar{2}01$)	42	20.99	6x20	2.433
Co($3\bar{3}01$)	58	20.16	4x20	2.591
Co($4\bar{4}01$)	78	20.39	3x20	2.524
Co($6\bar{6}01$)	114	20.22	2x20	2.543
Co($1\bar{1}00$)	20	20.12	12x20	2.286
Co($11\bar{2}0$)	18	21.16	12x12	2.504
Co($22\bar{4}1$)	196	20.20	4x11	2.543
Co($11\bar{2}1$)	98	20.01	4x12	2.579
Co($11\bar{2}2$)	52	20.72	3x14	2.627
Co($11\bar{2}3$)	36	20.81	11x13	2.656
Co($11\bar{2}4$)	54	20.32	3x14	2.663
Co($11\bar{2}6$)	20	20.11	8x16	2.622
Co($21\bar{3}0$)	148	20.24	3x12	2.508
Co($21\bar{3}1$)	148	20.15	2x13	2.557
Co($21\bar{3}2$)	148	20.05	2x13	2.600
Co($21\bar{3}3$)	150	20.05	2x15	2.626
Co($21\bar{3}4$)	152	20.05	2x14	2.649

SUPPORTING INFORMATION

Table S3. Information about the calculations of different *fcc* Co surfaces, including the number of layers for respective surface, as well as the size of each surface in terms of vertical distance between top and bottom atom of the slab (z_{max} - z_{min}), number of ***k***-points, and surface energy. For the Wulff construction we used the surface energies obtained for relaxed systems.

	# of layers	z_{max} - z_{min} (Å)	*k*-point sampling	Surface energy (J/m^2)
**Co(111)**	11	20.29	20x20	2.077
**Co(665)**	114	20.16	2x20	2.165
**Co(554)**	94	20.12	2x20	2.189
**Co(443)**	76	20.59	3x20	2.221
**Co(332)**	56	20.61	3x20	2.273
**Co(221)**	36	20.50	6x14	2.354
**Co(331)**	26	20.16	10x10	2.406
**Co(551)**	42	20.18	6x12	2.452
**Co(110)**	24	28.58	20x14	2.441
**Co(320)**	42	19.50	4x14	2.577
**Co(210)**	28	21.22	6x20	2.621
**Co(310)**	38	20.56	9x14	2.666
**Co(410)**	48	20.03	4x14	2.656
**Co(510)**	60	20.34	6x14	2.639
**Co(610)**	72	20.51	2x14	2.611
**Co(100)**	14	21.09	20x20	2.502
**Co(511)**	31	20.29	6x20	2.600
**Co(311)**	20	20.14	9x20	2.520
**Co(211)**	30	20.09	6x20	2.445
**Co(322)**	50	20.46	4x20	2.332
**Co(433)**	70	20.80	3x20	2.263
**Co(544)**	88	20.25	2x20	2.224
**Co(321)**	44	20.20	9x20	2.511
**Co(431)**	60	20.34	4x9	2.523
**Co(432)**	64	20.56	3x12	2.404
**Co(531)**	35	20.20	6x9	2.573
**Co(654)**	102	20.23	2x12	2.282
**Co(731)**	45	20.14	4x9	2.640
**Co(951)**	60	20.05	3x6	2.588

SUPPORTING INFORMATION

Table S4. Information about the calculations of different *bcc* Fe surfaces, including the number of layers for respective surface, as well as the size of each surface in terms of vertical distance between top and bottom atom of the slab (z_{max} - z_{min}), number of ***k***-points, and surface energy. For the Wulff construction we used the surface energies obtained for relaxed systems.

	# of layers	z_{max} - z_{min} (Å)	*k*-point sampling	Surface energy (J/m²)
**Fe(111)**	26	21.23	14x16	2.691
**Fe(665)**	71	20.26	3x17	2.671
**Fe(554)**	59	20.07	3x18	2.666
**Fe(443)**	92	19.97	4x18	2.654
**Fe(332)**	35	20.52	5x18	2.633
**Fe(221)**	44	20.29	9x15	2.629
**Fe(331)**	64	20.46	9x14	2.598
**Fe(551)**	102	19.82	4x14	2.548
**Fe(110)**	13	24.02	14x20	2.426
**Fe(320)**	52	20.02	6x20	2.532
**Fe(210)**	28	20.43	9x20	2.541
**Fe(310)**	24	20.59	6x20	2.515
**Fe(410)**	60	20.26	5x20	2.529
**Fe(510)**	38	20.54	4x20	2.529
**Fe(610)**	88	20.25	3x20	2.530
**Fe(100)**	24	32.56	20x20	2.486
**Fe(511)**	76	20.43	4x20	2.566
**Fe(311)**	48	20.06	6x19	2.604
**Fe(211)**	38	20.80	9x18	2.573
**Fe(322)**	60	20.26	6x17	2.647
**Fe(433)**	43	20.39	4x17	2.667
**Fe(544)**	108	20.06	3x17	2.672
**Fe(321)**	28	20.43	6x17	2.599
**Fe(431)**	38	20.54	5x16	2.582
**Fe(432)**	78	20.24	4x20	2.632
**Fe(531)**	86	20.34	4x17	2.601
**Fe(654)**	126	20.16	2x19	2.653
**Fe(731)**	110	20.09	3x18	2.587
**Fe(951)**	74	20.12	2x17	2.583

References

[1] N. Arulmozhi, G. Jerkiewicz, *Electrocatalysis* **2016**, *7*, 507–518.
[2] D. Esau, F. M. Schuett, K. L. Varvaris, J. Björk, T. Jacob, G. Jerkiewicz, *Electrocatalysis* **2020**, *11*, 1–13.
[3] G. Kresse, J. Furthmüller, *Phys. Rev. B* **1996**, *54*, 11169–11186.
[4] P. E. Blöchl, *Phys. Rev. B* **1994**, *50*, 17953–17979.
[5] J. P. Perdew, K. Burke, M. Ernzerhof, *Phys. Rev. Lett.* **1996**, *77*, 3865–3868.
[6] A. Hjorth Larsen, J. Jørgen Mortensen, J. Blomqvist, I. E. Castelli, R. Christensen, M. Dułak, J. Friis, M. N. Groves, B. Hammer, C. Hargus, et al., *J. Phys. Condens. Matter* **2017**, *29*, 273002.

Author Contributions

F.M.S. and D.E. carried out all the experiments, optimized the CAFF methodology and parameters for each metal, analysed the data, and wrote the first version of the manuscript with equal contributions. K.L.V and S.G. contributed to materials synthesis. J.B. carried out the DFT calculations of the Wulff construction under the guidance of T.J. and J.R. *T.J. and *G.J. conceived the experiments, supervised the project, analysed the results and contributed to the manuscript writing. All authors contributed to the discussion section and the finalization of the text and figures of the manuscript.
